# Supplementary material for: Impact of Coated Zinc Oxide Nanoparticles on Photosystem II of Tomato Plants
Source: Materials (Basel). 2023 Aug 26;16(17):5846. doi: 10.3390/ma16175846 (PMC10488754; doi:10.3390/ma16175846)
Supplement: Supplementary file 1 [file materials-16-05846-s001.zip › materials-2534545-supplementary.pdf]

materials

Article

Impact of Coated Zinc Oxide Nanoparticles on Photosystem II of Tomato Plants

Panagiota Tryfon [1], Ilektra Sperdouli [2], Ioannis-Dimosthenis S. Adamakis [3], Stefanos Mourdikoudis [4,5], Michael Moustakas [6,*], and Catherine Dendrinou-Samara [1,*]

Table S1. Definitions of the chlorophyll fluorescence parameters used in the experiments.

Parameter	Definition	Calculation
F*v*/F*m*	Maximum efficiency of PSII photochemistry	(F*m* − F*o*)/F*m*
Φ_{PSII}	Effective quantum yield of PSII photochemistry	(F*m*′ − F*s*)/F*m*′
Φ_{NPQ}	Quantum yield of regulated non-photochemical energy loss in PSII	F*s*/F*m*′ − F*s*/F*m*
Φ_{NO}	Quantum yield of nonregulated energy loss in PSII	F*s*/F*m*
F*v*′/F*m*′	Efficiency of open PSII centers	(F*m*′ − F*o*′)/F*m*′
F*v*/F*o*	Efficiency of the oxygen evolving complex (OEC) on the donor side of PSII	(F*m* − F*o*)/F*o*
ETR	Electron transport rate	Φ_{PSII} × PAR × c × abs, where PAR is the photosynthetically active radiation, c is 0.5, and abs is the total light absorption of the leaf taken as 0.84
q*p*	Photochemical quenching, representing the redox state of quinone A (Q_A), or in other words the fraction of PSII reaction centers in open state	(F*m*′ − F*s*)/(F*m*′ − F*o*′)
NPQ	Non-photochemical quenching reflecting the dissipation of excitation energy as heat	(F*m* − F*m*′)/F*m*′
EXC	Excess excitation energy	(1- q*p*) × F*v*′/F*m*′

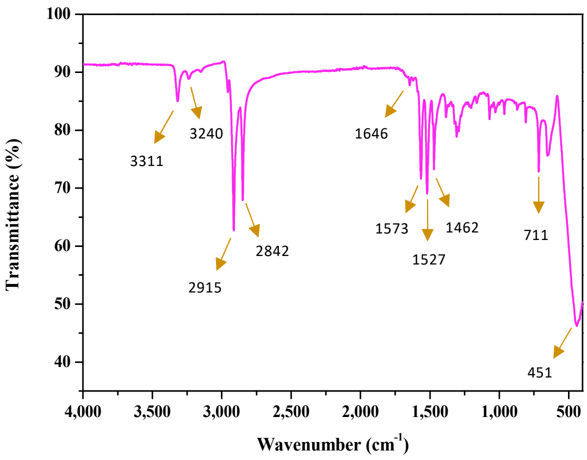

Figure S1. Fourier transform infrared spectroscopy (FT-IR) spectra of the ZnO@OAm NPs.

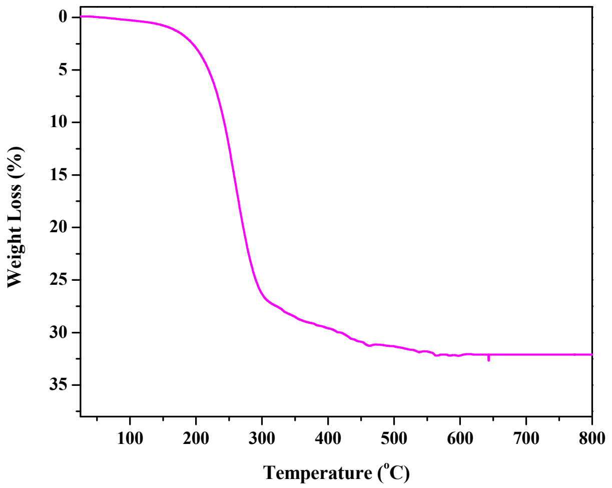

Figure S2. Thermogravimetric analysis curve of ZnO@OAm NPs with the main steps of organic coating weight loss.

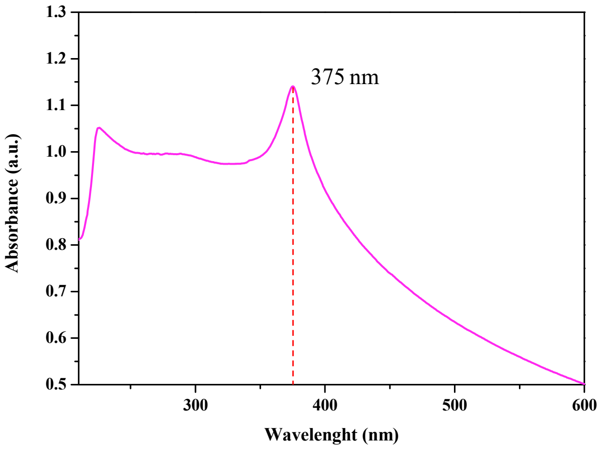

Figure S3. UV-Vis absorption spectrum of the ZnO@OAm NPs after preparation of NPs in a water-ethanol media.

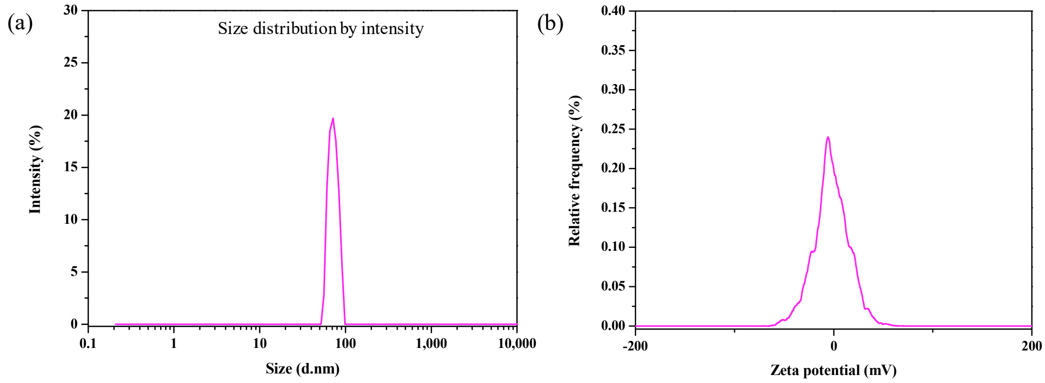

Figure S4. Size distribution **(a)** and ζ-potential **(b)** of the solvothermally prepared ZnO@OAm NPs.
